# Anterior cingulate is a source of valence-specific information about value and uncertainty

**DOI:** 10.1038/s41467-017-00072-y

**Published:** 2017-07-26

**Authors:** Ilya E. Monosov

**Affiliations:** Departments of Neuroscience and Biomedical Engineering, Washington University in St. Louis, St. Louis, MO 63110 USA

## Abstract

Anterior cingulate cortex (ACC) is thought to control a wide range of reward, punishment, and uncertainty-related behaviors. However, how it does so is unclear. Here, in a Pavlovian procedure in which monkeys displayed a diverse repertoire of reward-related, punishment-related, and uncertainty-related behaviors, we show that many ACC-neurons represent expected value and uncertainty in a valence-specific manner, signaling value or uncertainty predictions about either rewards or punishments. Other ACC-neurons signal prediction information about rewards and punishments by displaying excitation to both (rather than excitation to one and inhibition to the other). This diversity in valence representations may support the role of ACC in many behavioral states that are either enhanced by reward and punishment (e.g., vigilance) or specific to either reward or punishment (e.g., approach and avoidance). Also, this first demonstration of punishment-uncertainty signals in the brain suggests that ACC could be a target for the treatment of uncertainty-related disorders of mood.

## Abstract (web summary)

Rewards or punishments elicit diverse behavioral responses; however, the neural circuits underlying such flexibility are unclear. Here Monosov shows that this diversity could be supported by neurons in the anterior cingulate that represent expected value and uncertainty in a valence-specific manner.

## Introduction

Actions to maximize rewards and minimize threats or punishments are thought to be controlled by a region of the prefrontal cortex called the anterior cingulate cortex (ACC), but how this control is accomplished is unclear. Currently, there are several views of how ACC controls motivated behavior^1–9^. Some posit that the ACC neurons rank-order outcomes (such as rewards and punishments) by their utility or value. The resulting signed value signal from ACC is then read out by other brain areas to guide action towards the best possible option or outcome^2, 5–8, 10^. This theory posits that many single ACC neurons carry information about both rewards and punishments, and rank-order predictions of these outcomes according to their expected value. Another theory posits that ACC controls negative affect, threat, and pain-driven behaviors. This theory suggests that many ACC neurons ought to preferentially signal punishment-related information^11–14^. A third theory suggests that the ACC is primarily engaged in deploying attention to monitor task-performance and detect errors in our predictions and actions^15–22^. This theory predicts that many ACC neurons ought to be activated by salient, engaging, and cognitively demanding situations, irrespective of their valence. Finally, a fourth theory based on imaging studies in humans identifies ACC as a central hub for processing information about uncertainty and other exploration-related and learning-related variables, such as the value of foraging^23–27^. Several recent proposals suggest that ACC could support some or even many of these functions^1, 3, 4, 6, 7, 22, 27–36^, however how it could do so is unclear.

One explanation that could reconcile these different theories is that the ACC may contain multiple circuits that could separately process control-related variables, such as information about reward, punishment, and uncertainty. Such diversity within ACC would allow other brain areas to read out inputs from the ACC flexibly, either by combining or multiplexing information about reward, punishment, and uncertainty, or by processing this information separately in a valence-specific manner.

To assess how single ACC neurons signal reward, punishment, and uncertainty, single-neuron activity was recorded from ACC while monkeys experienced certain and uncertain predictions about rewards and punishments. These predictions were cued by well-learned single visual fractals that served as conditioned stimuli (CS). This approach isolates value and uncertainty signals in the activity of single ACC neurons and avoids choice-related or learning-related fluctuations in subjective value and uncertainty^37–42^.

The results suggest that the ACC contains groups of neurons that process appetitive, aversive, salient, and uncertain information, over short and long time scales. Particularly, many ACC neurons represented expected value and uncertainty in a valence-specific manner, signaling value or uncertainty of predictions about either rewards or punishments. Other neurons signaled information about rewards and punishments by displaying excitation to both, rather than excitation to one and inhibition to the other. These data caution against a unified view of ACC and suggest that the ACC contains multiple circuits that could contribute to the top-down control of a wide range of reward, punishment, and uncertainty-related internal states and actions in complementary but partly distinct ways.

## Results

### Diverse behaviors in response to rewards and punishments

**Fig. 1:**
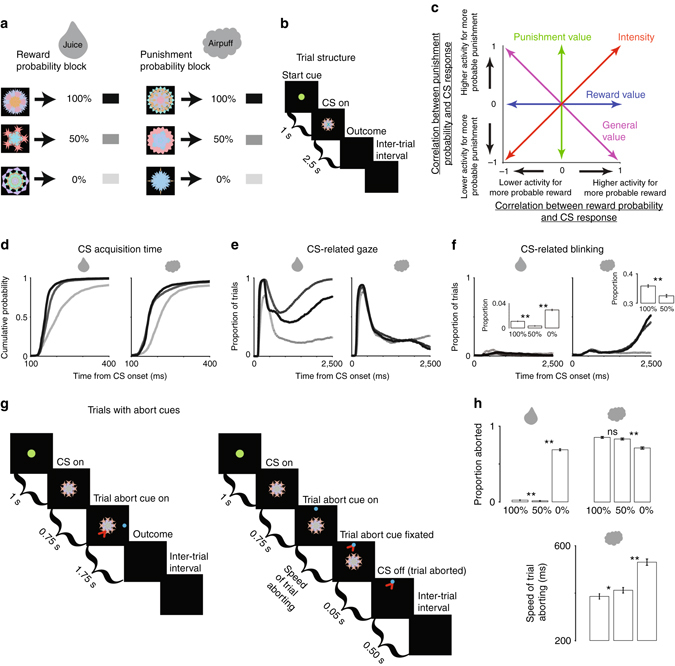
The reward-punishment behavioral procedure. **a** Monkeys experienced two types of distinct blocks of trials in which three visual fractal-conditioned stimuli (CSs) predicted rewards and punishments with 100, 50, and 0% chance. The reward block consisted of 12 trials in which reward was possible, and the punishment block consisted of 12 trials in which punishment was possible. **b** Structure of a single trial. CS could appear in the center (as shown) or peripherally, 10 degrees to the left or right of center. **c** Theoretical valence-coding strategies in the reward-punishment procedure. **d**–**f** Monkeys’ behaviors were motivated by reward, punishment, and uncertainty. **d** Cumulative probability functions of peripheral-CS gaze acquisition time. In reward or punishment block, the speed of CS acquisition was correlated with outcome probability (Spearman’s rank correlations; *p* < 0.01). **e** Proportion of trials the monkeys oriented to the location of peripheral CSs shown across the entire CS epoch for reward-block and punishment-block trials. During the last 1000 ms of the trial, the monkeys’ gaze was preferentially attracted to the 50% reward CS (*p* < 0.01; rank sum test). **f** Proportion of trials the monkeys blinked shown across the entire CS epoch for reward-block and punishment-block trials. *Insets* show mean proportion of time monkeys blinked during the last 500 ms of the CS epoch. **g** In distinct blocks we included an abort cue during approximately one half of the trials (Methods). Structure of trials with an abort cue. (**g**, *left*)—trials in which the monkeys did not abort (eye position is schematized by the red arrow); (**g**, *right*)—trials in which the monkeys aborted the trial. **h** Proportion of trials aborted in the reward block (*top-left*) and punishment block (*top-right*). The speed of trial aborting decreased with increasing punishment probability (*bottom*). The *black–gray* color legend for **d**–**f** are defined in **a**. *Error bars* indicate standard error. *Single asterisks* indicate significance at a 0.05 threshold and *double asterisks* indicate significance at 0.01 threshold

To test how single ACC neurons (n = 329) signal information about reward, punishment, and uncertainty, two monkeys were conditioned with an appetitive–aversive behavioral procedure that contained two separate contexts, or blocks. One block contained 12 trials in which three visual fractal objects (CS) predicted rewards (juice) with 100, 50, and 0% chance. The second block contained 12 trials in which three visual fractal CSs predicted punishments (air puffs) with 100, 50, and 0% chance (Fig. 1a, b). The monkeys did not have to fixate the CSs to complete the trial (Methods). The design was such that the 100% reward CS had the highest value in the reward block and the 0% punishment CS had the highest value in the aversive block. Hypothetical encoding strategies of reward and punishment predictions are shown in Fig. 1c.

Analyses across all recorded behavioral sessions showed that the monkeys understood the behavioral procedure. Their CS-related anticipatory mouth movements (e.g., licking of the juice spout) and anticipatory blinking were correlated to the probability of reward and punishment, respectively (Spearman’s rank correlations; *p* < 0.001; rho = 0.24 for licking; rho = 0.37 for blinking; Supplementary Fig. 1). While these anticipatory responses were related to the expected value of the CSs, other behaviors, such as gaze, were driven by the absolute expected value of the CSs (often called motivational intensity or salience^43^) and by outcome uncertainty. Particularly, the monkeys’ gaze was initially drawn to the CSs associated with the more probable outcome (Fig. 1d), irrespective of valence. Target acquisition times during all the 100 and 50% CS trials were faster when compared with the 0% trials, within either reward or punishment block (*p* < 0.001; rank sum tests; single trials from no-abort cue blocks from all behavioral sessions = 31,838 trials). Within the reward block, target acquisition times were faster during 100% reward CS trials than during 50% reward CS trials (*p* < 0.01; rank sum tests).

Later in the trial the monkeys’ gaze was most strongly attracted towards the 50% reward CS (Fig. 1e; rank sum test; *p* < 0.001; measured during the last 1 s of the trial). We could not reliably observe CS-related gaze behavior during the last second of the punishment-predicting CSs because the gaze signal was quenched by defensive blinking (Fig. 1f).

To study punishment, it is crucial to verify that the outcome or unconditioned stimulus is aversive. It could be that blinking behaviors depicted in Fig. 1f reflect conditioning, but not aversion. To address this issue, we utilized distinct reward and punishment blocks that contained abort cues during one-half of the trials (Methods). This paradigm is a type of active avoidance used to test the aversiveness of cues, outcomes, and contexts in humans and experimental animals^44–48^. If monkeys made a saccade to the abort cue, the trial was aborted (Fig. 1g). Because these reward and punishment blocks alternate, the optimal reward-driven strategy would be to rapidly abort every trial in which the 100 and 50% reward CSs were not presented. In contrast, the monkeys’ aborting behaviors were influenced by reward, punishment, and uncertainty. In the punishment block, monkeys actively aborted punishment-predicting CSs more often than the 0% punishment CS, confirming that the air puff was an aversive outcome (Fig. 1h). While initially 100% punishment CS was associated with faster target acquisition than 0% CS (suggesting that it was more motivationally salient because it strongly attracted gaze; 43), later in the trial (when abort cues were presented), monkeys aborted the 100% CS faster than 50% and 0% punishment CSs. The data show that the monkeys’ motivation to abort was positively related to the probability of punishment.

Also, monkeys aborted 50% reward CSs less than 100% reward CS trials (though, note that the proportion of aborted trials during either 100 or 50% reward CSs was extremely low). This decrease in abort error rate is consistent with the observations in Fig. 1e that the 50% reward CS captured attention (despite having a lower expected value than 100% reward CS), reducing the number of saccades to the location of the abort-cue.

In sum, the behavioral data suggest that monkeys utilized different representations (or encoding strategies) of rewards and punishments to influence their behaviors.

Next, we asked if the ACC contains distinct representations of rewards and punishments or if ACC neurons encode rewards and punishments with a common currency, such as a general value signal (Fig. 1c). The locations of ACC neuronal recordings are shown in Supplementary Fig. 2 and match the locations of neuronal recordings in previous studies of macaque ACC^5, 9, 32, 49, 50^.

### Many ACC cells signal value of either reward or punishment

The neuronal recordings revealed both that many ACC neurons represent expected value in a valence-specific manner, displaying greatest sensitivity to the probability of either rewards or punishments; and also that many ACC neurons prefer uncertain CSs in a valence-specific manner, often displaying preference for either reward or punishment uncertainty.

**Fig. 2:**
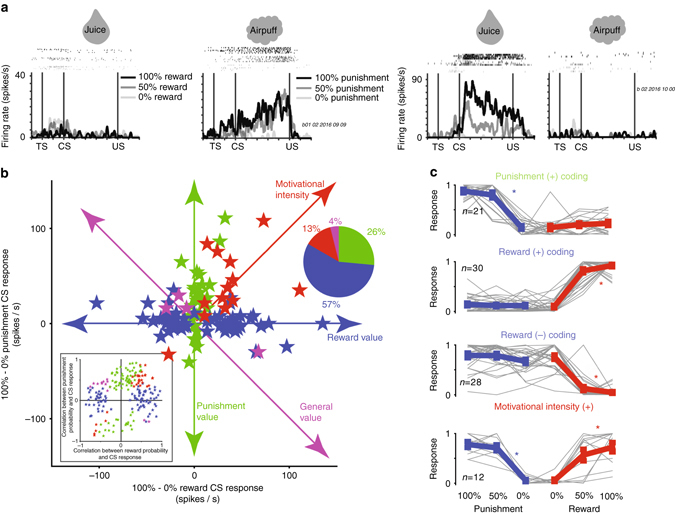
Many ACC neurons signal outcome probability in a valence-specific manner. **a** Two single ACC neurons, one that signals punishment probability (*left*) and one that signals reward probability (*right*). **b** Scatter plot shows the difference in CS responses for 100 and 0% CSs in the reward block (*x*-axis) vs. difference in CS responses for 100 and 0% CSs in the punishment-block (*y*-axis). *Green*—neurons displayed significant differences between 100 and 0% CSs in the punishment block only, *blue*—neurons that displayed significant differences between 100 and 0% CSs in the reward block only, *red*—neurons that displayed significant differences between 100 and 0% CSs in both blocks and both differences were either positive or negative, and *purple*—neurons that displayed significant differences between 100 and 0% CSs in both blocks and the differences were of different sign. Colors in pie chart (*inset*) correspond to colors in scatter plot and in Fig. 1c. Uncertainty-selective neurons are studied separately in Fig. 3. (**b**, *inset*) Correlation coefficients of all recorded neurons that displayed significant correlations with either reward or punishment probabilities, or both. Significance of each correlation was tested by 10,000 permutations (*p* < 0.05). **c** Single neuron responses (*gray*) shown separately for four major groups of outcome probability coding neurons in the scatter plot in **b**. Single neurons’ CS responses were normalized to the maximum CS response; from 0 to 1. *Red asterisks* indicate that the neurons’ responses varied significantly across the CSs within the reward block (Kruskal–Wallis test; *p* < 0.01) and displayed significant correlation with outcome probability (Spearman’s rank correlation; *p* < 0.01); *blue asterisks* indicate that the neurons’ responses varied significantly across the CSs within the punishment block (Kruskal–Wallis test; *p* < 0.01) and displayed significant correlation with outcome probability (Spearman’s rank correlation; *p* < 0.01)

To summarize the valence-encoding strategies of ACC neurons, correlation analyses were peformed for each recorded neuron (*n* = 329), which assessed the relationship of CS responses and outcome probability (Methods; Supplementary Fig. 3 and Supplementary Table 1). The correlation coefficients are shown in Fig. 2b-inset. Most neurons that displayed significant correlations with outcome probability (Spearman’s rank correlation; statistical threshold: *p* < 0.05) did so in a valence-specific manner, for either reward or punishment probability.

Two valence-specific example neurons are shown in Fig. 2a. The first neuron displayed greatest excitation to punishment-predicting CSs and did not differentiate among 100, 50, and 0% reward CSs. The second neuron displayed greatest excitation to reward-predicting CSs (strongest to 100% reward) and did not differentiate among 100, 50, and 0% punishment CSs. To assess if such specific valence coding strategies were common among ACC neurons, we visualized the differences in neuronal responses for predictions of good outcomes and bad outcomes in the reward and punishment blocks (Fig. 2b). These analyses confirmed that many value-coding ACC neurons display valence-specific responses (Fig. 2).

Notably, other studies showed that within reward loss or reward gain trials, distinct ACC neurons signaled predictions and deliveries of reward gains and losses^7, 28, 51, 52^. Our data replicate these studies. Among reward-value neurons, many were excited or inhibited by increasing probabilities of rewards (Fig. 2c), signaling positive or negative reward values, respectively. In contrast, among the punishment-value neurons, most were excited by increasing probabilities of punishments.

Also, these ACC neurons did not display-trial-by-trial correlations with conditioned responses; see Supplementary Table 2.

The second most common valence coding strategy seen among ACC neurons signaled the motivational intensity or unsigned value of the CSs (red in Fig. 2). Few neurons showed a generalized value signal that rank-ordered the reward or punishment predictions according to their expected value (100 > 50 > 0% rewards and 0 > 50 > 100% punishments; Fig. 2). The time courses of neuronal responses among these neuronal types are shown in Supplementary Figs. 4–5.

Following 50% CSs, many ACC neurons discriminated between outcome deliveries and omissions. Many of these prediction error responses also tended to be valence specific. For example, distinct ACC neurons signaled reward omissions, reward deliveries, punishment omissions, and punishment deliveries (Supplementary Fig. 3). Interestingly, the value of the CSs (during the CS epoch) and their associated outcomes were often signaled by different neurons (Supplementary Fig. 3). To summarize, the data thus far suggest that ACC neurons can signal the values of predictions and deliveries of rewards and punishments through the activity of distinct populations of neurons.

**Fig. 3:**
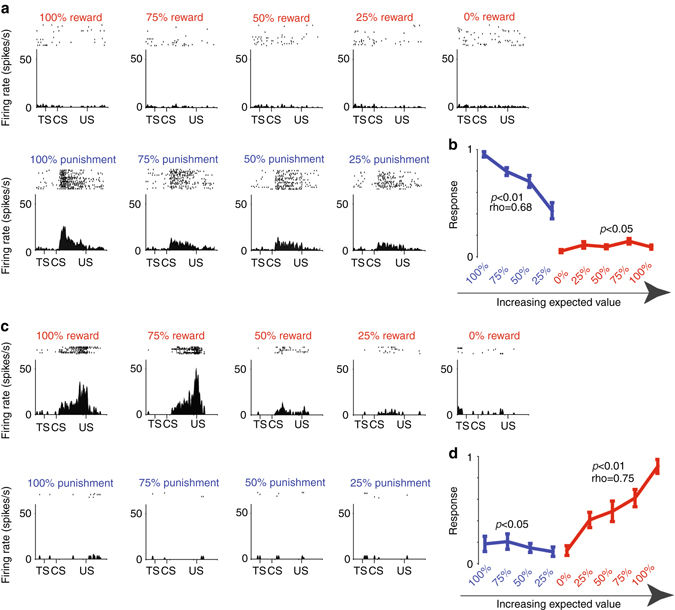
Valence specificity in a single-block reward/punishment procedure. **a** The activity of a single punishment-sensitive ACC neuron shown separately for the nine CSs in the single block reward/punishment procedure. **b** The average activity of 16 punishment-enhanced neurons in ACC. **c** The activity of a single reward-sensitive ACC neuron shown separately for all nine CSs. **d** The average activity of nine reward-enhanced neurons in ACC. Spearman’s rank correlations of the average responses with reward and punishment probabilities are indicated in **b** and **d**. *Error bars* denote standard error. Classification of neurons as punishment-enhanced value coding neurons and reward-enhanced value coding neurons were the same as in Fig. 2 (Methods). Before averaging, single neurons’ CS responses were normalized to the maximum CS response; from 0 to 1 (same as in Fig. 2c). Red—activity in reward trials, blue—activity in punishment trials

ACC neurons are known to discriminate among different behavioral tasks^33^ and contexts^53^, and integrate task-related information over long time scales^31, 54^. Hence, it was important to assess if ACC contains valence-specific neurons in a behavioral procedure in which reward and punishment CSs are presented in the same context or block of time. To this end, in an additional control procedure, Monkey B was conditioned with nine distinct visual fractal CSs that predicted reward with 100, 75, 50, 25, and 0% chance and punishment with 100, 75, 50, and 25% chance. The monkey displayed conditioned responses that suggested that it understood the meaning of the CSs. The monkey’s licking and blinking behaviors were correlated with the probability of reward and punishment, respectively (Supplementary Fig. 6A). Among 95 ACC neurons recorded in this procedure, 16 were selectively enhanced by increases in the probability of punishment, but were insensitive to changes in the probability of reward. An example of one such neuron and their average responses are shown in Fig. 3a, b. Oppositely, 9/95 neurons were enhanced by increases in the probability of reward, but were insensitive to changes in the probability of punishment. An example of one such reward-specific value-coding neuron and their average activity are shown in Fig. 3c, d. Therefore, in the blocked reward-punishment behavioral procedure (Fig. 1) and in the single-block control procedure, the ACC contained neurons that signaled reward and punishment values in a valence-specific manner. Consistent with this observation, population coding strategies (summarized by plotting correlation coefficients of correlations that assessed the relationship of neuronal activity and probabilities of either reward or punishment) were qualitatively similar across the blocked and the single-block behavioral procedures (Fig. 2b; Supplementary Fig. 6B).

**Fig. 4:**
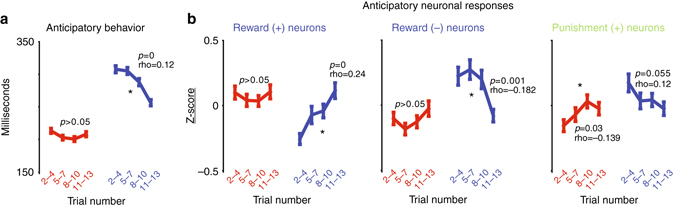
Monkeys and ACC neurons anticipate the nearing of their preferred block. **a** Reward and punishment blocks of 12 trials were separated into four sub-blocks. The blocks were separated relative to the monkeys’ knowledge about which block they were in. For example, the reward block starts after the first reward CS trial after which the monkeys knew they were in the reward block. It ends during the first punishment CS trial (because the monkeys had not yet seen the punishment CS). Hence, the block trial numbers in this figure are numbered 2 through 13. The average times of when monkeys foveated the trial start cue (relative to the time of the trial start cue presentation) are shown for the four sub-blocks in the reward block (*red*, *left*) and aversive block (*blue*, *right*). The results of a Spearman’s rank correlations assessing the relationship of sub-block number and speed of orienting to the trial start cue are indicated above each block. *Asterisk* highlights when a correlation was significant. Error bars denote standard error. **b** Responses of the three groups of valence-specific neurons identified in Fig. 2 during the last 500 ms of the trial start cue epoch. Conventions are the same as in **a**. In all four plots, the data in the reward block (*red*) differed from the data in the punishment block (*blue*; rank sum test; *p* < 0.05)

In the two-block appetitive/aversive procedure, monkeys and ACC neurons were highly sensitive to the nearing of their preferred context over many trials (Fig. 4). To show this, the analyses were concentrated on the behavioral and neuronal responses to the trial start cue. Though trial start cues were presented 3.5 s before the trial’s outcome would be experienced by the monkeys, their anticipatory orienting behavior (the duration it took them to foveate the trial start cue) was significantly faster in the reward vs. punishment block (Fig. 4a). Also, during the punishment block the speed of target acquisition changed as a function of trial number, decreasing as the reward block neared (Fig. 4a). Similarly, reward and punishment-sensitive neurons signaled the nearing of their preferred blocks. Reward-sensitive neurons displayed gradual and systematic changes in the aversive block in anticipation of the reward block (Fig. 4b). And, punishment-sensitive neurons displayed systematic changes in the reward block in anticipation of the aversive block (Fig. 4b).

Therefore, ACC neurons can signal valence specifically or non-specifically (Fig. 2). And, valence-specific neurons can signal the nearing of their preferred contexts or events over long time scales (Fig. 4).

### Representation of reward and punishment uncertainty in ACC

Next, we assess if and how single ACC neurons encode uncertainty. In contrast with the hypothesis that ACC encodes economic or general common currency values, some recent neuroimaging studies have highlighted the ACC as a central hub for processing outcome uncertainty and guiding behaviors aimed to reduce this uncertainty. However, single neuron evidence for uncertainty processing in ACC has been missing.

**Fig. 5:**
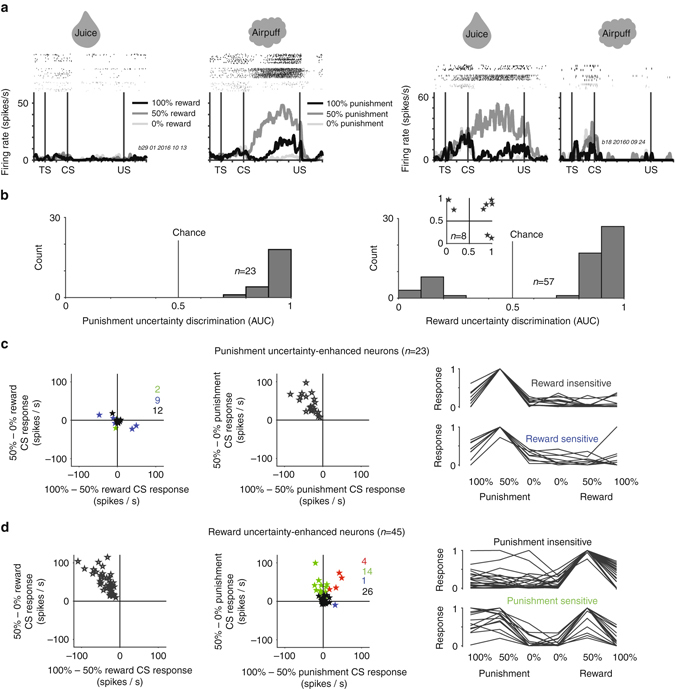
Distinct coding of reward and punishment uncertainty by many ACC neurons. **a** Two single ACC neurons, one that signals punishment uncertainty (*left*) and one that signals reward uncertainty (*right*). **b** Single neuron discrimination indices of punishment and reward uncertainty-selective neurons (*left* and *right*). *Inset* shows discrimination of eight neurons that displayed uncertainty selectivity in both reward (*x*-axis) and punishment blocks (*y*-axis). Discrimination indices were obtained by ROC analyses (Methods). Values below 0.5 indicate selective suppression, indices above 0.5 indicate selective enhancement. **c** Punishment uncertainty-enhanced neurons in the reward block (*left-scatter plot*) and punishment block (*right-scatter plot*). Scatter plots show, for individual neurons, the differences between 50 vs. 100% CS responses (*x*-axis) and 50 vs. 0% CS responses (*y*-axis). *Red stars*, neurons showing significant differences in CS response in both 50–100% comparison and 50–0% comparison. *Green stars*, neurons showing significant differences in only 50–0% comparison. *Blue stars*, neurons showing significant differences in only 100–50% comparison. *Black stars*, neurons showing no significant differences. Single neuron and average CS responses (*right*) for two major groups of punishment uncertainty enhanced neurons (*black and blue stars on the left*). **d** Reward uncertainty-enhanced neurons. Conventions are the same as in **c**

We found 88 uncertainty selective neurons in the ACC (Methods), some of which responded for punishment uncertainty. An example neuron is shown in Fig. 5a (*left*). This neuron did not respond selectively to reward-predicting CSs or reward outcomes. In the punishment block, the neuron was most strongly activated by 50% punishment CS until the trial outcome. Among uncertainty selective neurons, 23/88 neurons were selectively excited by punishment uncertainty but not reward uncertainty (Fig. 5b; Supplementary Fig. 7). Zero were selectively inhibited by punishment uncertainty (Fig. 5b, c). Among the punishment-uncertainty neurons, nine neurons displayed significant variability (Kruskal–Wallis test; Methods) among the reward block CSs (Fig. 5c) but there was no obvious trend for positive or negative reward value coding among them. These results show that some ACC neurons signal punishment uncertainty in a selective manner, and that a minority of them can also multiplex this uncertainty signal with information about rewards.

Consistent with the observation that ACC can signal task-related information in a valence-specific manner (Fig. 2), we also found neurons that preferred reward uncertainty but not punishment uncertainty (Fig. 5a, b). An example neuron is shown in Fig. 5a-right. This neuron responded to the 50% reward CS and maintained tonic firing until the time of the trial-outcome (reward or no reward). It was not modulated by punishment predicting CSs. 45/88 uncertainty selective neurons were excited by reward uncertainty, while 12/88 uncertainty selective neurons were inhibited. Some reward uncertainty excited neurons also carried information about punishment probability; Fig. 5d and Supplementary Fig. 7. Among these, almost all were enhanced by increased probability of punishment. Finally, a minority of uncertainty selective neurons (8/88) were selective for both reward and punishment uncertainty (Fig. 5b).

Like all other populations of uncertainty or risk-coding neurons, ACC uncertainty neurons discriminated between 100 and 0% CSs in their preferred block^37, 55–58^. Within the aversive block, 9/23 punishment uncertainty neurons responded more to 100 than 0% punishment CSs, and 3/23 responded more to 0 than 100%. 22/45 reward uncertainty enhanced neurons responded more to 100 than 0% reward CSs, and 0/45 responded more to 0 than 100% reward CSs.

Outcome deliveries following uncertainty elicit prediction errors^43, 59, 60^. However, the majority of ACC uncertainty neurons did not signal prediction errors (Supplementary Fig. 8). This observation provides further evidence for the notion that within ACC, there may be distinct groups of neurons tracking predictions and outcome-related or feedback-related prediction errors (Supplementary Fig. 3).

In sum, ACC can signal uncertainty about either rewards or punishments in a valence-specific manner or to multiplex information about uncertainty and value in multiple manners (Supplementary Fig. 9). Also, prediction errors following uncertain epochs were often signaled by distinct populations of neurons.

Uncertainty can arise due to variability in outcomes or due to the possibility of making an error. For example, in our behavioral procedure aborting a reward-associated CS is an error because it reduces the probability of reward on that trial to 0. Though we observed few such errors (Fig. 1), the anticipation of the abort cue resulted in increases of overt attention towards the reward-possible CSs and the activity of uncertainty neurons in ACC (Supplementary Fig. 10). Therefore, ACC uncertainty neurons are sensitive to uncertainty arising due to internal processing (that increases attention^27^) as well as due to uncertain stimulus-outcome associations.

Recent studies show that several subcortical brain regions in the septum and the striatum contain populations of neurons that signal the graded level of reward uncertainty^56, 57^. Because these brain regions receive inputs from the ACC^61, 62^, an important question is, do ACC reward uncertainty neurons also signal graded levels of uncertainty and reward size? To answer this question, ACC reward enhanced uncertainty neurons were recorded while monkeys participated in the reward probability/reward amount behavioral procedures used in our previous studies^55–57, 63^. The reward-probability block contained five objects associated with five probabilistic reward predictions (0, 25, 50, 75 and 100% of 0.25 ml of juice). The reward-amount block contained five objects associated with certain reward predictions of varying reward amounts (0.25, 0.1875, 0.125, 0.065 and 0 ml). The expected values of the five CSs in the probability block matched the expected value of the five CSs in the amount block. The block design was used to remove the confounds introduced by risk-seeking related changes in subjective value processing of the CSs^37, 55–57^.

**Fig. 6:**
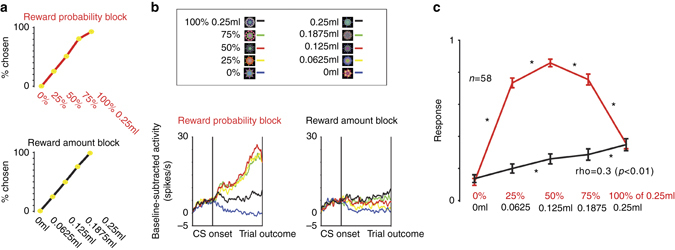
Reward uncertainty ACC neurons are sensitive to the level of uncertainty. **a** Monkeys’ choice preference between CSs associated with different reward amounts and probabilities. Choice percentage of a single reward probability CS (*x*-axis) vs. all other reward probability CSs (*top*). Choice percentage of a single reward amount CS vs. all other reward amount CSs (*bottom*). Data are compiled from a data set of 13407 choice trials. **b** Average responses of 58 reward uncertainty-enhanced ACC neurons in the reward-probability block (*left*) and reward amount block (*right*). **c** Average normalized responses of the same neurons in the probability (*red*) and amount (*black*) CSs. *Asterisks* indicate differences between adjacent CSs (*p* < 0.05; paired sign-rank test). The result of a Spearman’s rank correlation assessing the relationship of neuronal firing and reward amount in the reward amount block is indicated below. Before averaging, single neuronal responses were normalized to the maximum CS response, from 0 to 1, across the ten different CSs. *Error bars* indicate standard error

Consistent with our previous reports using the same procedure, after conditioning, monkeys choices rank ordered the CSs in either block according to their expected values (Fig. 6a, see refs ^55–57, 63^) indicating they understood the meanings of the CSs. After training, 58 ACC reward uncertainty enhanced neurons were identified and studied in the single CS reward probability/reward amount behavioral procedure.

The online identification of reward uncertainty neurons was the same as in our previous studies^56, 57, 63^. The average response of ACC reward uncertainty enhanced neurons is shown in Fig. 6b, c. During the reward-probability block, the neurons responded most strongly to the most uncertain CS (50% CS), more weakly to 25 and 75% CSs. The same neurons displayed the weakest response to the certain CSs (0 and 100%). In the reward amount block, their responses encoded reward size in a roughly linear manner, displaying highest firing for the greatest CS associated with the greatest reward sizes (Fig. 6c; rho = 0.3; *p* < 0.001; Spearman’s rank correlation; *n* = 58). These data indicate that, on average, ACC reward-uncertainty neurons signal the level of reward uncertainty and can also signal reward amounts in a roughly linear manner (Fig. 6b).

## Discussion

It has been proposed that the ACC exerts top–down control on a wide-range of reward, punishment, and uncertainty-related internal states and action selection processes. But how single ACC neurons encode reward, punishment, and uncertainty has remained unclear. Here, in a Pavlovian procedure in which monkeys displayed a diverse repertoire of reward, punishment, and uncertainty-related behaviors, many ACC neurons represented expected-value and uncertainty in a valence-specific manner, signaling value or uncertainty of either rewards or punishments. Also, other ACC neurons signaled information about rewards and punishments by displaying excitation to both, rather than excitation to one and inhibition to the other.

These results are mostly incompatible with several theories of ACC. First, though the ACC did contain neurons that were selectively activated by punishment predictions, there was no indication that the majority of ACC neurons exclusively signaled aversive or noxious information. Second, the data are inconsistent with the notion that many single ACC neurons encode a general value or common currency signal that generalizes across appetitive and aversive contexts (such as a theoretical economic value signal). Very few ACC neurons displayed positive relationships with reward probability and negative relationships with punishment probability (or vice versa).

In contrast, the data are consistent with the hypothesis that the ACC contains multiple circuits that have the capacity to distinctly process control-related and motivation-related variables, such as information about reward, punishment, and uncertainty. This diversity in information coding strategies could allow other brain areas to read-out inputs from the ACC flexibly, either by combining or multiplexing information about reward, punishment, and uncertainty; or by processing such information separately, to mediate many internal states and actions^1–4, 22, 31, 33, 34, 49, 53, 64^, such as those that are enhanced by both rewards and punishments (e.g., vigilance), and those specific to either reward or punishment (e.g., approach and avoidance).

This study demonstrates a novel selective punishment uncertainty signal in the brain and will therefore provide new opportunities to study internal states, such as anxiety and depression, which are thought to be strongly driven by persistent uncertainty about bad outcomes^25, 65–68^.

Punishment uncertainty neurons were often found in more anterior and ventral regions of the bank of the ACC while reward uncertainty neurons were found in all areas, but were particularly common in the dorsal extent of the ACC. Since the dorsal and ventral regions of the dorsal ACC have differences in their projection patterns to the striatum, amygdala, cortex, and brain stem^62, 69–71^, one possibility is that reward and punishment uncertainty are processed by distinct circuits involving somewhat different (but overlapping) areas of ACC. This conjecture is further supported by a series of experiments in which we identified neurons in two anatomically distinct networks that signal information about reward uncertainty but not punishment uncertainty: the septal-basal forebrain network and the striatal-pallidal network^55–57, 63^. Both of these networks receive inputs from and send direct and indirect inputs to the ACC, and neither displayed consistent coding of punishment uncertainty.

Previous work has identified that the ACC may be important for monitoring decision uncertainty and decision outcomes^72^ and for information seeking^4, 73^. It will be important to assess how different groups of neurons identified in this study, and throughout the subcortical uncertainty-related network, contribute to those functions.

When rewards and punishments are bundled into a single stimulus (or option) that monkeys can choose to approach or avoid, neurons in the pregenual cingulate cortex (an area that is anatomically distinct from but related to regions studied in this article) were either correlated with approach behavior or avoidance^74^. Interestingly, the avoidance-related neurons were also more active when the monkeys’ decision reaction times were slow. The authors suggested that this effect may be related to the subjective conflict between the reward and punishment within a particular bundle^74^. Along similar lines, Ebitz and Platt found that in a saccade task, distractor-induced conflict enhanced the activity of many neurons located within the ACC^22^. Here, it was found that the possibility of an abort cue presentation during high-valued trials (that monkeys rarely aborted) enhanced the activity of ACC uncertainty-sensitive neurons. In these trials, the abort cue was an aversive distractor to which saccades ought not to be made. One interesting hypothesis is that task-conflict, the increased probability of action-performance errors and of reward prediction errors (following uncertainty) modulate the same groups of ACC neurons.

ACC neurons display selectivity for contexts and tasks^33, 53^. Compatible with those observations was the finding that valence sensitive neurons predicted and anticipated the nearing of their preferred context over long time-scales. Also, on average, the CS responses of many reward and punishment-sensitive neurons did not consistently differ across blocks in which abort cues may be presented vs. blocks in which the abort cues were never presented, suggesting that ACC neurons may have the capacity to adjust their responses relative to the values of the possible predictions within a given context or block of trials.

Anterior regions of area 14c within the macaque ventromedial prefrontal cortex (vmPFC) and the dorsal raphe nucleus serotonin neurons, like ACC, display a capacity for context and valence specificity^38, 75, 76^ by tonically and phasically signaling the valence of blocks or contexts. One possibility that now requires serious investigation is that serotonin could play a crucial regulatory role in valence and context-specific behavioral selection through raphe-to-prefrontal projections to the ACC and vmPFC.

This study has several limitations. Rewards and punishments often elicit diverse and distinct behavioral states^77, 78^. This makes the dissociation of a valence-specific value signal from an action-value signal difficult. Though ACC neurons, like neurons in the orbitofrontal cortex^42^, did not display trial-by-trial correlations with conditioned responses, they likely influence action by increasing or decreasing motivation in a valence, context, and action-specific manner. Also, because one of the aims of this study was to ask if ACC contained uncertainty selective neurons, uncertainty that was unrelated to the CS was minimized. For example, the behavioral procedures did not include choice trials in which uncertainty or risk can come about due to many factors^25, 37, 79, 80^. It will be important to understand the time course and dynamics of reward and punishment-related signals in ACC during choice behavior, particularly in economic decision making tasks in which rewards and punishments are combined into single choice options.

Identifying neuronal mechanisms that facilitate flexible control of action and learning toward rewards and away from punishments remains a central pursuit of systems neuroscience. Here, we demonstrate a remarkable diversity and valence specificity of ACC neurons that may in part provide a neuronal substrate for such capacity. The data suggest that the ACC has multiple distinct and cooperative functions in behavioral control related to the expectation and receipt of reward, punishment, and their uncertainty.

## Methods

### General procedures

Three adult male rhesus monkeys (*Macaca mulatta*) were used for the neurophysiology experiments (1—Monkey B, 2—Monkey Z, and 3—Monkey R). All procedures conformed to the Guide for the Care and Use of Laboratory Animals and were approved by the Washington University Institutional Animal Care and Use Committee. A plastic head holder and plastic recording chamber were fixed to the skull under general anesthesia and sterile surgical conditions. The chambers were tilted laterally by 35° and aimed at the anterior cingulate and the anterior regions of the basal ganglia. After the monkeys recovered from surgery, they participated in the behavioral and neurophysiological experiments.

### Data acquisition

While the monkeys participated in the behavioral procedure we recorded single neurons in the anterior cingulate. The recording sites were determined with 1 mm-spacing grid system and with the aid of MR images (3T) obtained along the direction of the recording chamber. This MRI-based estimation of neuron recording locations was aided by custom-built software (PyElectrode). Single-unit recording was performed using glass-coated electrodes (Alpha Omega). The electrode was inserted into the brain through a stainless-steel guide tube and advanced by an oil-driven micromanipulator (MO-97A, Narishige). Signal acquisition (including amplification and filtering) was performed using Alpha Omega 44 kHz SNR system. Action potential waveforms were identified online by multiple time-amplitude windows with an additional template matching algorithm (Alpha-Omega). Neuronal recording was restricted to single neurons that were isolated online. Neuronal and behavioral analyses were conducted offline in Matlab (Mathworks, Natick, MA).

Eye position was obtained with an infrared video camera (Eyelink, SR Research). Behavioral events and visual stimuli were controlled by Matlab (Mathworks, Natick, MA) with Psychophysics Toolbox extensions. Juice, used as reward, was delivered with a solenoid delivery reward system (CRIST Instruments). Juice-related licking was measured and quantified using previously described methods^63^. Airpuff (~35 psi) was delivered through a narrow tube placed ~6–8 cm from the monkey’s face.

### Behavioral procedures

The reward-punishment behavioral procedure consisted of two alternating blocks of trials: a reward block and a punishment block. In the reward block, three visual fractal CS were followed by a liquid reward (0.4 ml of juice) with 100, 50, and 0% chance, respectively. In the punishment block, three visual fractal CSs were followed by an air puff with 100, 50, and 0% chance, respectively. One block consisted of 12 trials with fixed proportions of trial types (each of the three CSs appears four times during each block). The inter-trial-intervals (ITIs) ranged from ~2–6 s.

Each trial started with the presentation of a trial-start cue at the center. The trial-start cue disappeared after 1 s and one of the three CSs was presented pseudo randomly (the CS could appear in three locations: 10 degrees to the left or to the right of the trial-start cue or in the center). After 2.5 s, the CS disappeared, and the outcome (juice, aifpuff, or nothing) was delivered. The monkeys were not required to fixate.

Abort cues were presented during one half of the trials in distinct blocks (abort-blocks) in one of three locations 10 degrees away from the CS. Abort blocks contained distinct visual fractals as CSs. Typically, a recording session contained the following repeating block structure: reward (abort) block, punishment (abort) block, reward block, punishment block, reward block, punishment block.

An additional single block reward-punishment behavioral procedure was used to study the activity of ACC in monkey B. The trial structure was the same as in the two-block procedure. Here, nine visual fractals served as CSs that predicted reward with 100, 75, 50, 25, and 0% chance and punishment with 100, 75, 50, and 25% chance. No abort cues were presented in this procedure.

To study if reward uncertainty-sensitive ACC neurons signal the level of reward uncertainty and reward size, a five reward-probability and reward-amount procedure was used. This procedure consisted of two blocks, a reward-probability block and a reward-amount block. The trial structure was detailed in White and Monosov, 2016 (see ref. ^57^). Each trial started with the presentation of a green trial-start cue at the center. The monkeys had to maintain fixation on the trial-start cue for 1 s; then the trial start cue disappeared and one of the CSs was presented pseudo randomly. After 2.5 s, the CS disappeared, and juice (if scheduled for that trial) was delivered. The reward-probability block contained five visual fractal objects CSs associated with five probabilistic reward predictions (0, 25, 50, 75 and 100% of 0.25 ml of juice). The reward-amount block contained five objects associated with certain reward predictions of varying reward amounts (0.25, 0.1875, 0.125, 0.065 and 0 ml). Each block consisted of 20 trials with fixed proportions of trial types (each of the five CSs appears four times in each block). Monkeys R, B, and Z participated in this procedure.

In separate experimental sessions, the monkeys’ choice preference was tested for the CSs. This procedure has been detailed in our previous studies^56, 63^. Each trial started with the presentation of the trial-start cue at the center, and the monkeys had to fixate it for 0.5 s. Then two CSs appeared 10 degrees to the left and right. The monkeys had to make a saccade to one of the two CSs within 5 s and fixate it for at least 750 ms. Then the unchosen CS disappeared, and after 1 s the outcome (associated with the chosen CS) was delivered, and the chosen CS disappeared. If the monkey failed to fixate one of the CSs, the trial was aborted and all stimuli disappeared. The trials were presented pseudo randomly, so that a block of 180 trials contained all possible combinations of the 10 CSs four times. Monkeys R, B, and Z participated in this procedure.

### Data processing and statistics

Spike-density functions were generated by convolving spike times with a Gaussian filter (*σ* = 100 ms). All statistical tests were two-tailed. For comparisons between two task conditions for each neuron, we used a rank-sum test, unless otherwise noted. For comparisons between two task conditions across the population average, we used a paired signed-rank test, unless otherwise noted. *p* < 0.05 with Bonferroni correction was used as a threshold for these tests unless otherwise noted.

All correlation analyses were Spearman’s rank correlations. The significance of the correlation analyses (threshold: *p* < 0.05, unless otherwise noted) was tested by 10,000 permutations^38, 56, 57, 63^.

CS responses were measured in a single time window that started 100 ms from the CS onset until the outcome. Outcome responses were measured in a single 500-ms window that started 50 ms following the outcome delivery. To normalize task-event-related responses, we subtracted baseline activity (the last 500 ms of the inter-trial interval) from the activity during the task-event related measurement epoch.

A neuron was defined as CS responsive if it displayed variance across CSs either in the reward or punishment block (Kruskal–Wallis test, *p* < 0.01). The statistical identification of uncertainty neurons was detailed previously^38, 56, 57, 63^. As before, a neuron was defined as uncertainty sensitive if its CS responses varied across the three possible outcome predictions in either block (Kruskal–Wallis test, *p* < 0.01) and if its response to the uncertain CS (50%) was significantly stronger or weaker than its responses to both 100 and 0% CSs (two-tailed rank-sum test; *p* < 0.05; Bonferroni corrected). Therefore, a neuron could be uncertainty-sensitive in reward block, punishment block, or both.

To calculate receiver operating characteristic (ROC) that assessed neuronal discrimination of value and uncertainty, we compared spike counts during the CS epoch of two conditions. The analysis was structured so that area under curve values >0.5 indicate that the response for the value or uncertainty was a selective enhancement, while values <0.5 indicate that the response for the value or uncertainty was a selective suppression.

Trial-by-trial correlations of single unit activity and conditioned responses (Supplementary Table 2) were performed so as to minimize the influence of global task correlations. For each neuron type, the neurons’ preferred CS was chosen (e.g., reward uncertainty enhanced neurons’ 50% reward CS epochs were analysed), and the relationship of spiking activity (mean spike count) and the magnitude of conditioned responses during that CS epoch was tested by a Spearman’s rank correlation. Similar results were obtained if partial correlations were performed across all CS conditions in which spiking activity and conditioned responses were z-scored within each CS condition before the correlation was performed. For the trial-by-trial correlations assessing firing rate during the CS epoch and the subsequent ITI pupil dilation, the pupils were assessed in a 0.50 s window, 1.5 s following the outcome. For each neuron, object selectivity was assessed by comparing its responses to two distinct visual fractals that conveyed the neuron’s preferred outcome prediction (e.g., reward value enhanced neurons’ responses for two visual fractals that conveyed 100% reward).

Supplementary Fig. 9 visualizes how uncertainty and value-coding strategies co-occur in single neurons across the reward and punishment blocks. Each neuron is represented by a vector of 4 value and uncertainty discrimination indices ranging from 0 to 1. Value discrimination indices were obtained by ROC analyses that compared 100 and 0% CS responses. Uncertainty discrimination indices were obtained by ROC analyses that compared 50 and 100% CS responses. If a neuron did not pass the uncertainty sensitivity test (described above), the result of the uncertainty ROC analysis was set to 0.5. If a neuron was uncertainty-sensitive in one of the blocks (i.e., reward or punishment blocks), its value index was set to 0.5 in that block. The data were organized by hierarchical clustering on Euclidean distance utilizing Ward’s minimum variance method. Because clustering is a data mining method, here it is utilized to organize the matrix of neurons for qualitative observation. The results are interpreted as visual confirmation for multiple value and uncertainty coding strategies in ACC, not as evidence for discrete and stable clusters (e.g., robust to noise or increases or decreases in neuron number).

### Data availability

Data supporting the findings of this study are available within the article and its Supplementary Information Figures or from the authors on request.

## Electronic supplementary material

Supplementary Information : Supplementary Figures and Supplementary Tables
