# Supplementary material for: Anterior cingulate is a source of valence-specific information about value and uncertainty
Source: Nat Commun. 2017 Jul 26;8:134. doi: 10.1038/s41467-017-00072-y (PMC5529456; doi:10.1038/s41467-017-00072-y)
Supplement: Supplementary file 1 — : Supplementary Figures and Supplementary Tables [file 41467_2017_72_MOESM1_ESM.pdf]

File name: Supplementary Information
Description: Supplementary Figures and Supplementary Tables

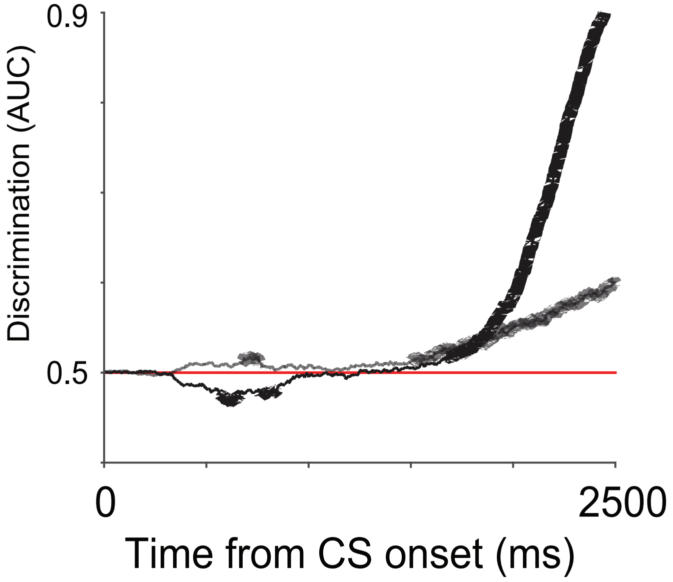

Supplementary Figure 1 **– Expression of aversive conditioned responses in two monkeys.** The results of an ROC analysis is shown in time comparing blinking during 100% and 0% CS punishment trials for Monkey 1 (black) and Monkey 2 (gray). Thicker lines indicate time points at which there was significant difference between 100% and 0% trials (rank sum tests; $p<0.01$ with Bonferroni correction for 2500 multiple comparisons for each millisecond in the CS epoch). Though both monkeys' blinking was strongly correlated with the probability of air puffs (Spearman's rank correlations, $p<0.01$), the difference between 100% and 0% CS trials was greater in Monkey 1. ROC analysis was structured so that receiver-operating characteristic area values > 0.5 indicate that the blinking in the 100% CS trials was greater than 0% CS trials; values < 0.5 indicate that the blinking in the 100% CS trials was less than 0% CS trials. AUC, area under ROC curve. AUC – area under ROC curve.

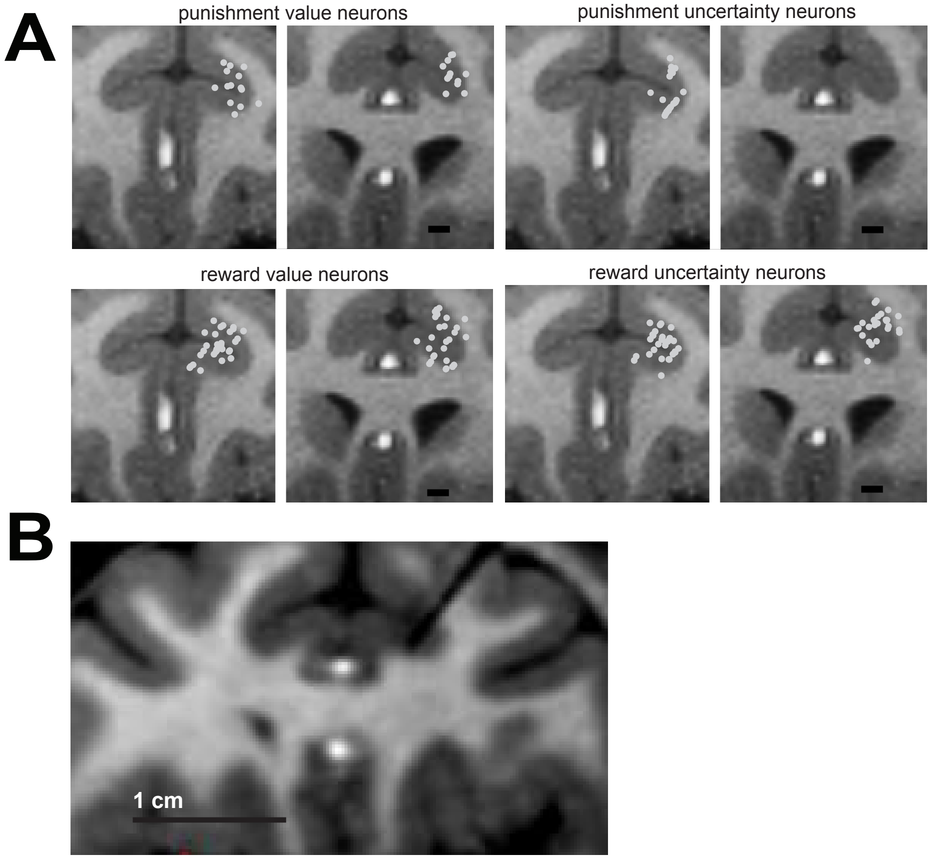

***Supplementary Figure 2* – Estimated locations of reward value, punishment value, reward uncertainty, and punishment uncertainty ACC neurons**. (**A**) The recording range was 6 to 17 mm anterior to the center of the anterior commissure (AC), and also 3 neurons sampled 3 mm anterior to the AC were included in this study (which did not change any of the results). The anterior section on the left is 15 mm from the center of the AC and includes neurons recorded 17mm to 12 mm from the center of the AC. The posterior section on the right is 11 mm from the center of the AC, and includes the neurons sampled in the posterior extent of our recordings. A notable observation is the qualitative difference in the location of reward uncertainty and punishment uncertainty neurons. Punishment uncertainty neurons were found in the most anterior extent of our recordings and their locations were often within the ventral regions of the bank of the ACC, while reward uncertainty neurons were often found dorsally and in general more widely throughout the area of the neuronal recordings. (**B**) A coronal T1 magnetic resonance image confirming a recording location of a punishment uncertainty neuron in the ACC. The image was acquired with a tungsten electrode (FHC) at the recording location within ACC. The electrode's shadow is the black line whose tip is in ACC.

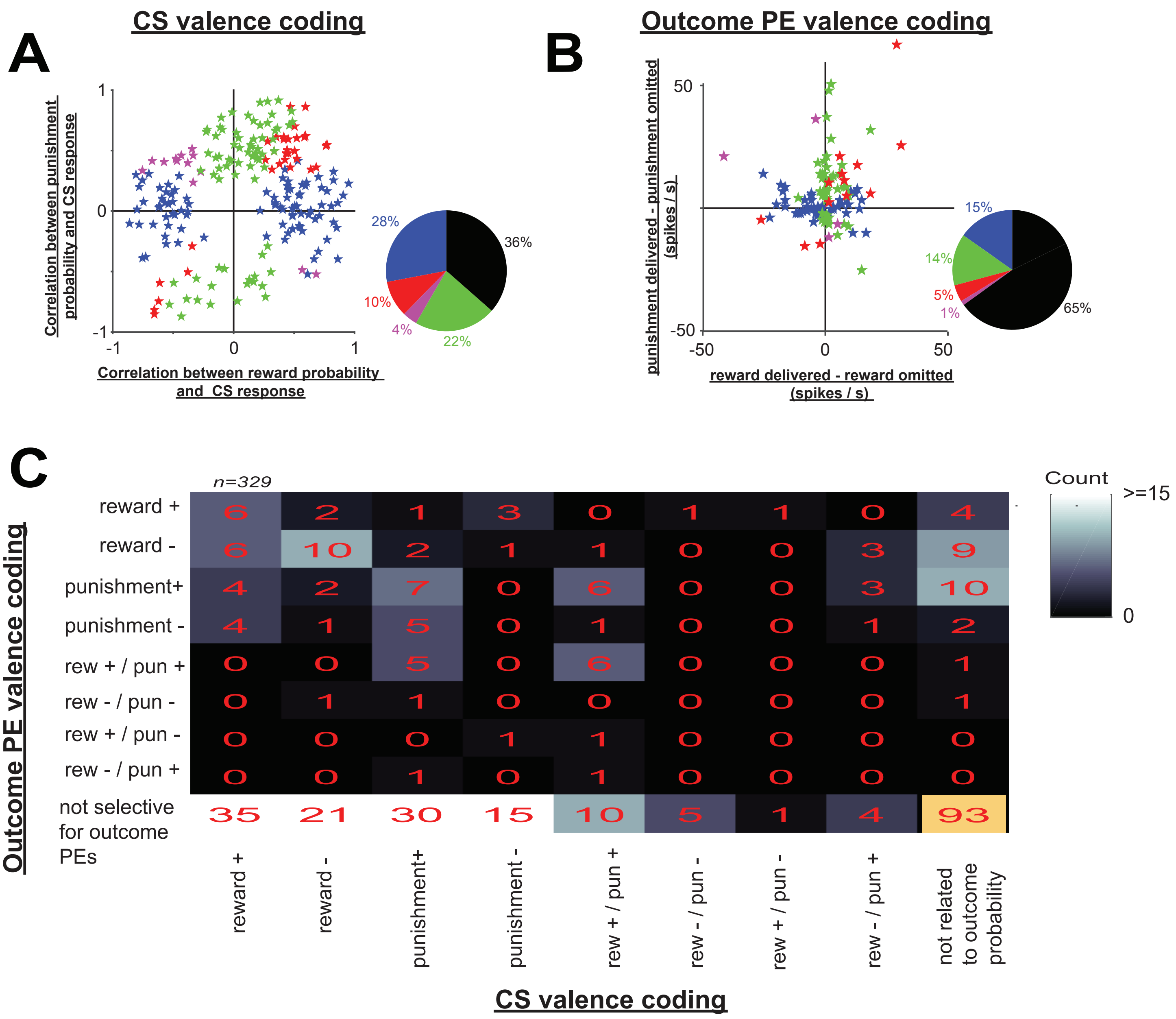

***Supplementary Figure 3* – Coding of reward and punishments: CS responses and outcome prediction errors.** (**A**) Significant correlation coefficients assessing the relationship of single neurons' responses and reward and punishment probability during the CS epoch. Significance of each correlation was tested by 10,000 permutations (Spearman's rank correlations; p<0.05). (**B**) Significant single neuron response differences between reward deliveries versus omissions following 50% reward CSs (x-axis), and differences between punishment deliveries versus omissions following 50% punishment CSs (y-axis). Significance was assessed by a Wilcoxon rank sum test (p<0.05). Colors in scatter plots and pie charts in **A-B** are the same as in Figure 2; black indicates percentage of neurons that did not reach significance. (**C**) Counts of neurons displaying significant outcome PE coding (y-axis; determined from **B**) and significant correlation coefficients from **A (x-axis).** On the y-axis, (+) denotes that the outcome-related activity was significantly higher when the outcome was delivered versus not delivered, (-) denotes that the outcome-related activity was significantly higher when the outcome was not delivered versus delivered. On the x-axis, the (+/-) are the signs of the significant correlation coefficients in **A**.

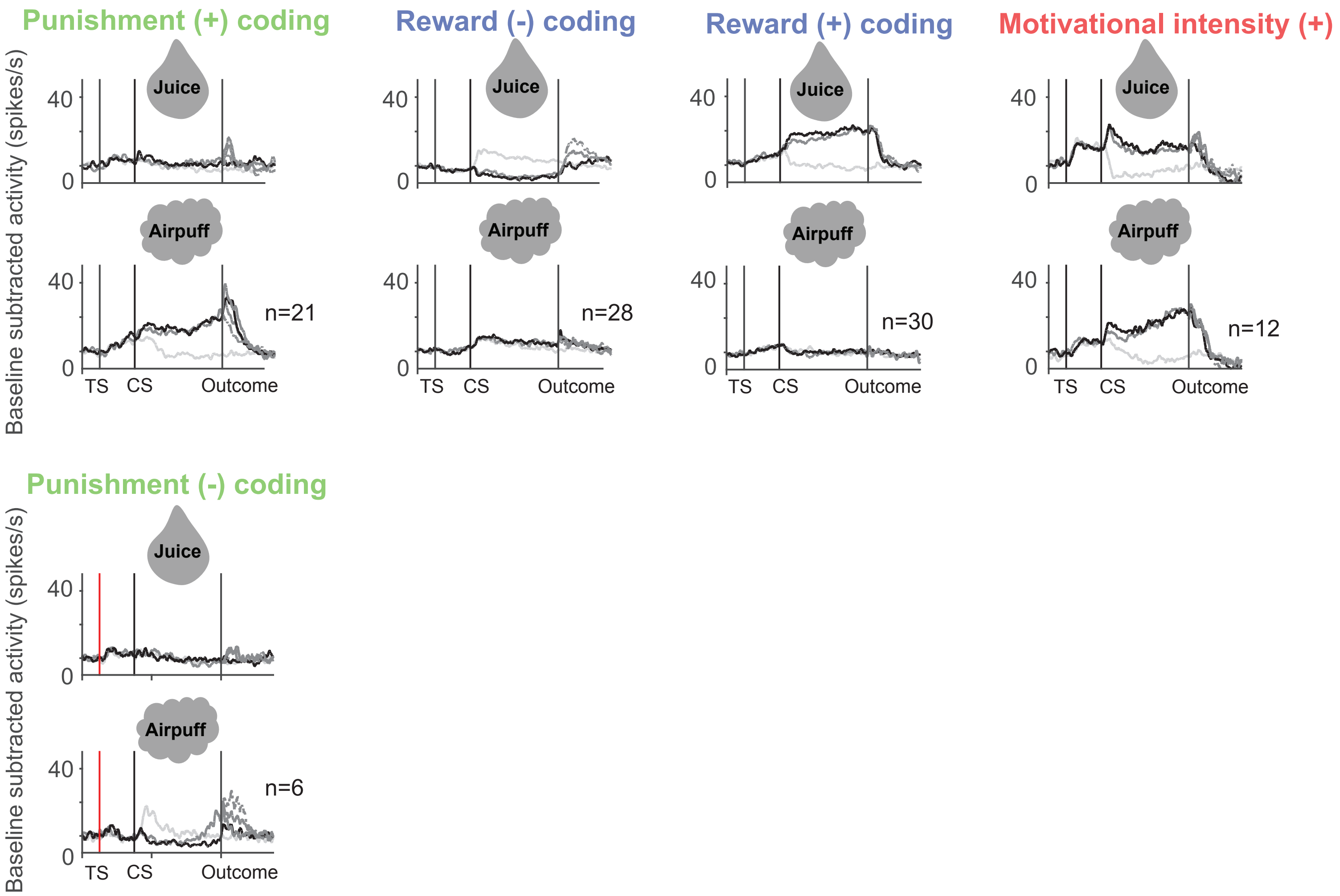

***Supplementary Figure 4* – Neuronal activity of outcome-probability value coding neurons.** Their activity in the reward (top) and punishment (bottom) blocks shown separately for 100, 50, and 0% CSs. Darkest traces show activity in 100% CS trials, lightest traces show activity in 0% CS trials (this is the same gray-black color scheme as in Figure 1). Dotted lines (after the outcome) represent outcome omissions. The neuronal classifications are the same as in Figure 2C.

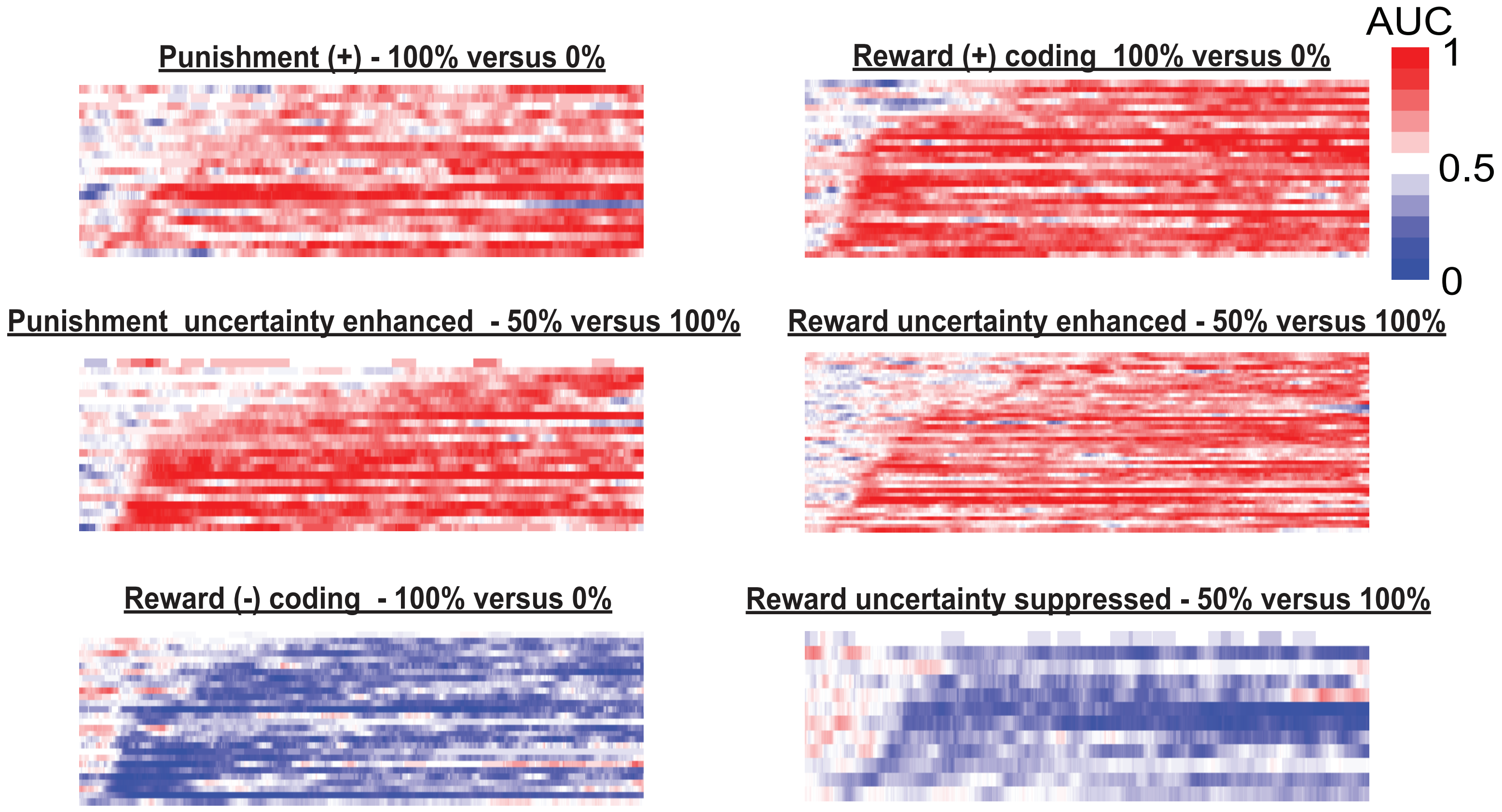

***Supplementary Figure 5* – Time courses of value and uncertainty discrimination in single neurons.** For each group of neurons (labeled above the heat maps), a receiver-operating characteristic (ROC) analysis was performed that compared spike density functions from CS onset to the time of outcome (2.5 seconds). Each line represents the results of this analysis for a single neuron during the entire CS epoch. For the value coding neurons the time course of value discrimination was assessed by an ROC analysis that compared 100% versus 0% CS trials in their preferred block. For uncertainty neurons, the time course of uncertainty discrimination was assessed by an ROC analyses that compared 50% versus 100% CS trials in their preferred block. ROC analyses were structured so that receiver-operating characteristic area values > 0.5 indicate that the activity in the 100% CS trials was greater than 0% CS trials for value neurons, and that 50% was greater than 100% CS trials for the uncertainty neurons; values < 0.5 indicate that the activity in the 100% CS trials was less than 0% CS trials for value neurons, and that 50% was less than 100% CS trials for the uncertainty neurons. AUC, area under ROC curve.

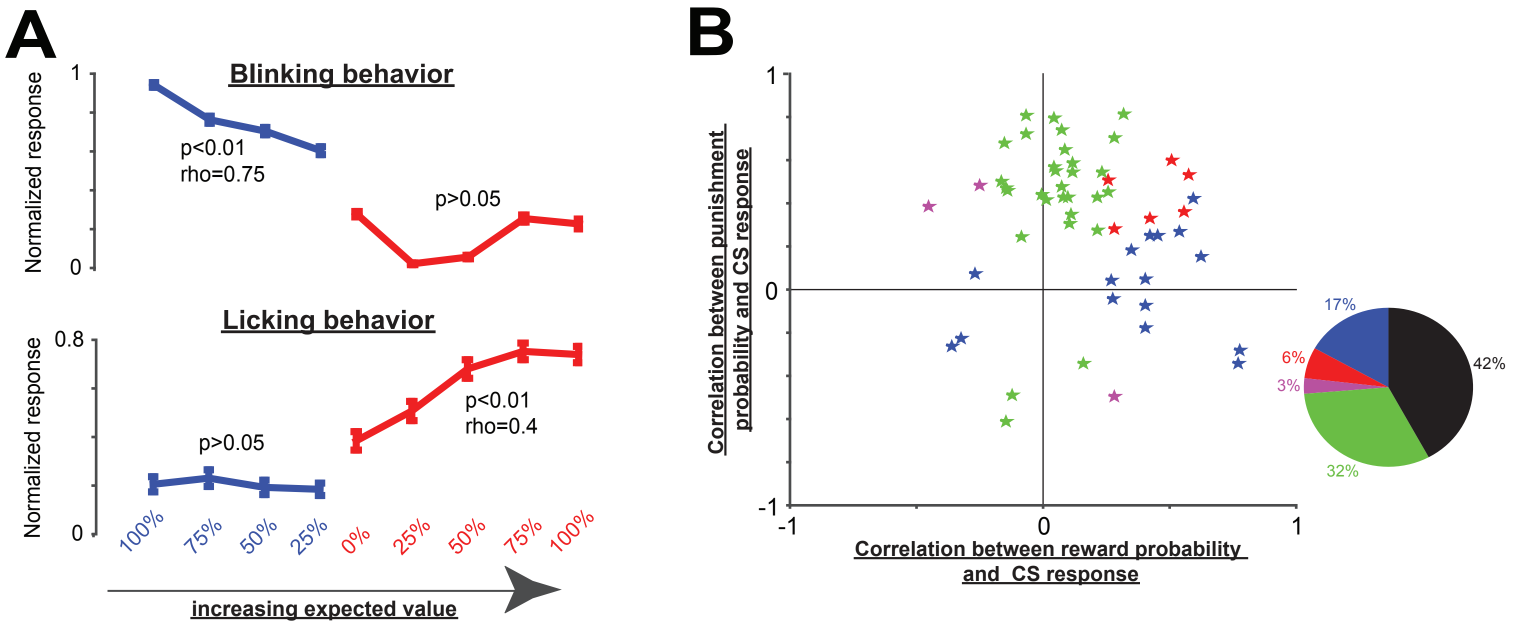

***Supplementary Figure 6* – Single block reward / punishment procedure.** (**A**) Conditioned responses to the presentation of 100, 75, 50, 25, 0% reward CSs and 100, 75, 50, 25% punishment CSs within a single block. Blinking (top) was correlated to the probability of punishment (rho=0.75; p<0.01), but not to the probability of reward (p>0.05). Though, as in Figure 1, blinking was least prevalent during reward uncertain conditions. Licking (bottom) was correlated to the probability of reward (rho=0.4; p<0.01), but not to the probability of punishment (p>0.05). Here, the behavioral CS responses were normalized to the maximum CS response; from 0 to 1. (**B**) Significant correlation coefficients of single neurons with the probability of reward and punishment (same format as Supplementary Figure 3A). Pie chart shows the percentages of neurons across the 95 recorded neurons during the single block reward / punishment procedure.

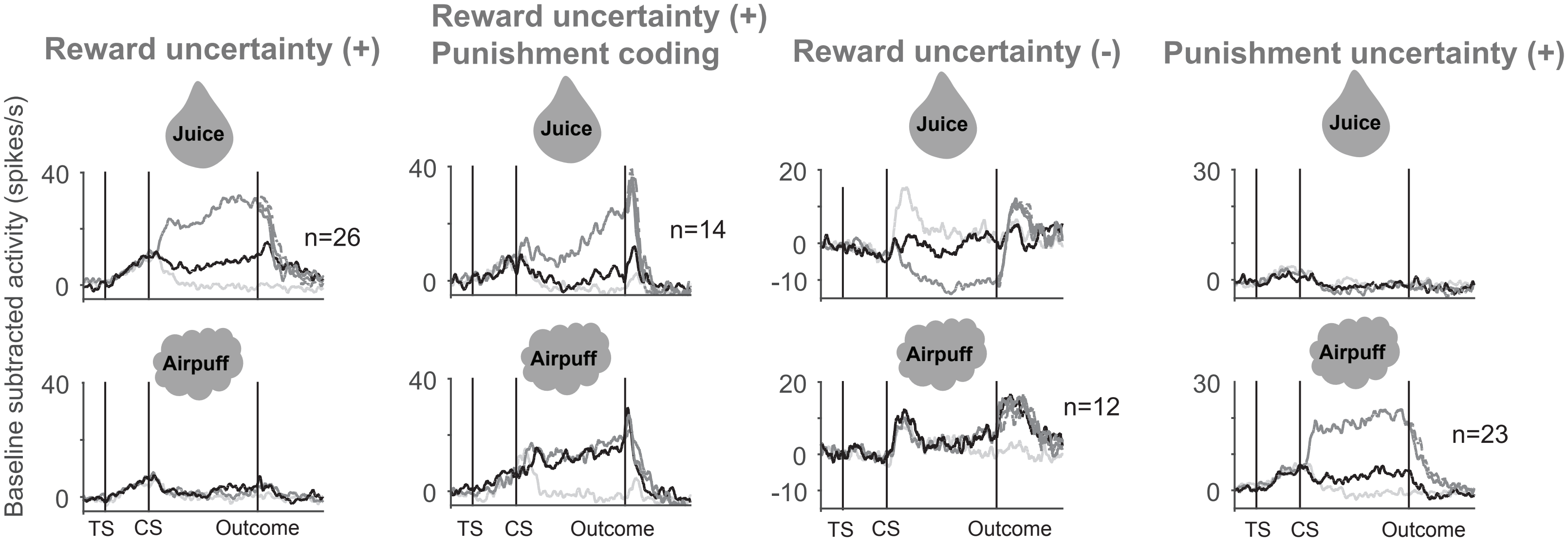

Supplementary Figure 7 **– Neuronal activity of uncertainty selective neurons.**
Conventions are the same as in Supplemental Figure 4.

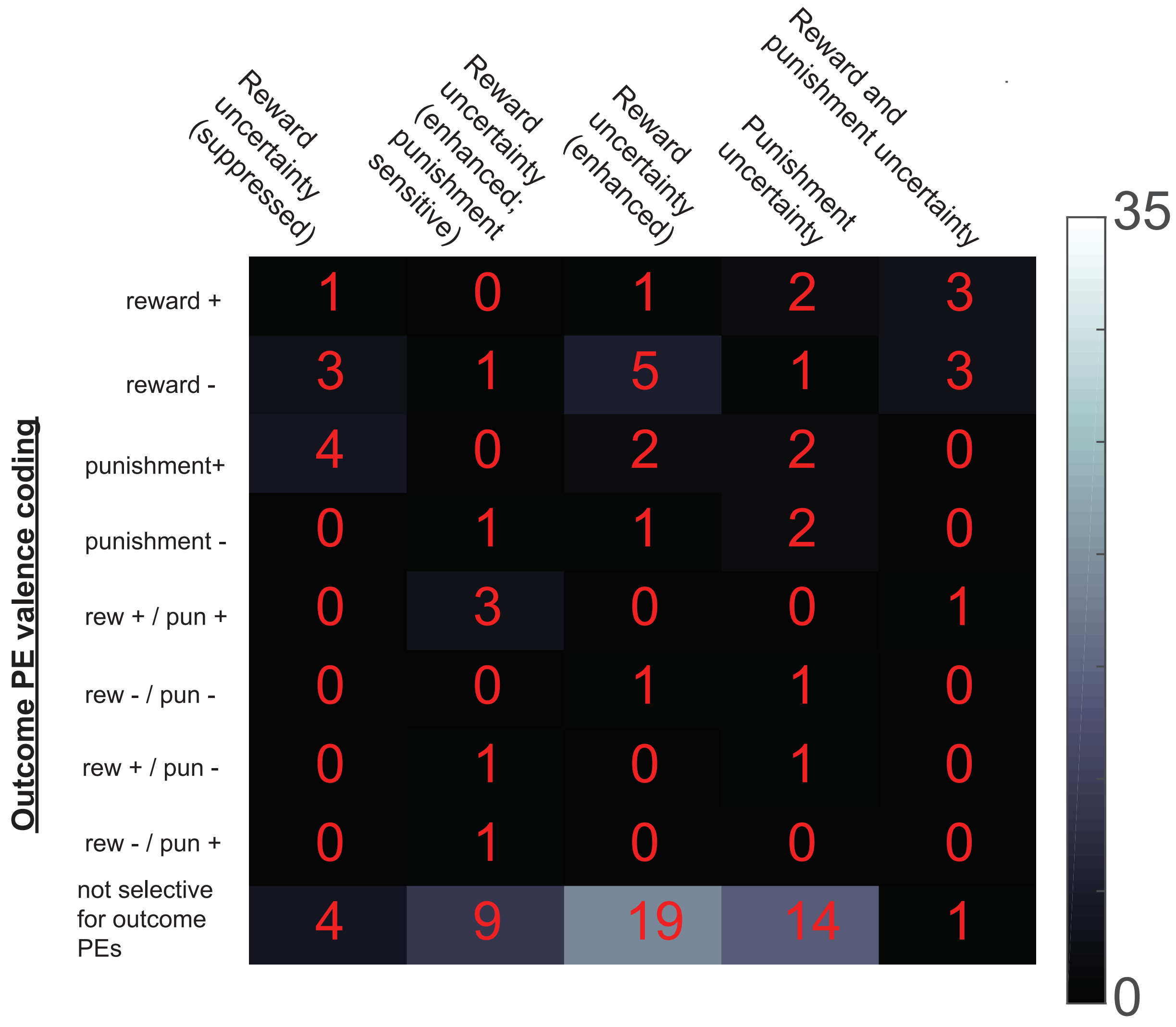

***Supplementary Figure 8* – Neuronal counts of uncertainty neurons displaying outcome prediction error coding.** The data are separated into groups of uncertainty neurons (x-axis). Format is the same as in Supplementary Figure 3.

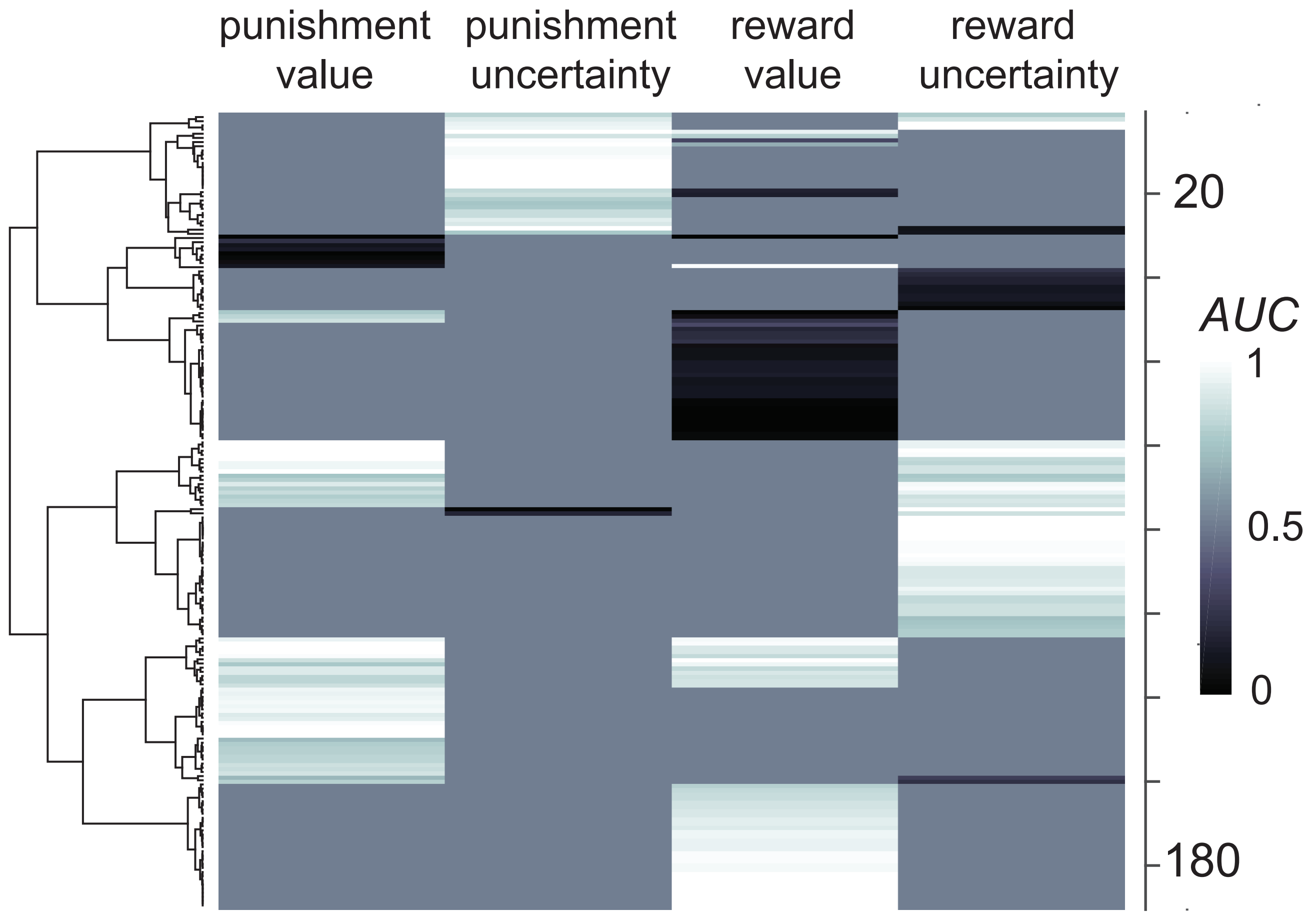

***Supplementary Figure 9* – ACC neurons uncertainty and value coding.** A visual summary of uncertainty and value sensitivity of all ACC neurons that displayed variance across the CSs (Kruskal Wallis test; p<0.01). Each neuron (shown as a row) contributes either an uncertainty or value discrimination index in each block. Matrix in this plot was organized by an unsupervised hierarchical clustering algorithm (Methods).

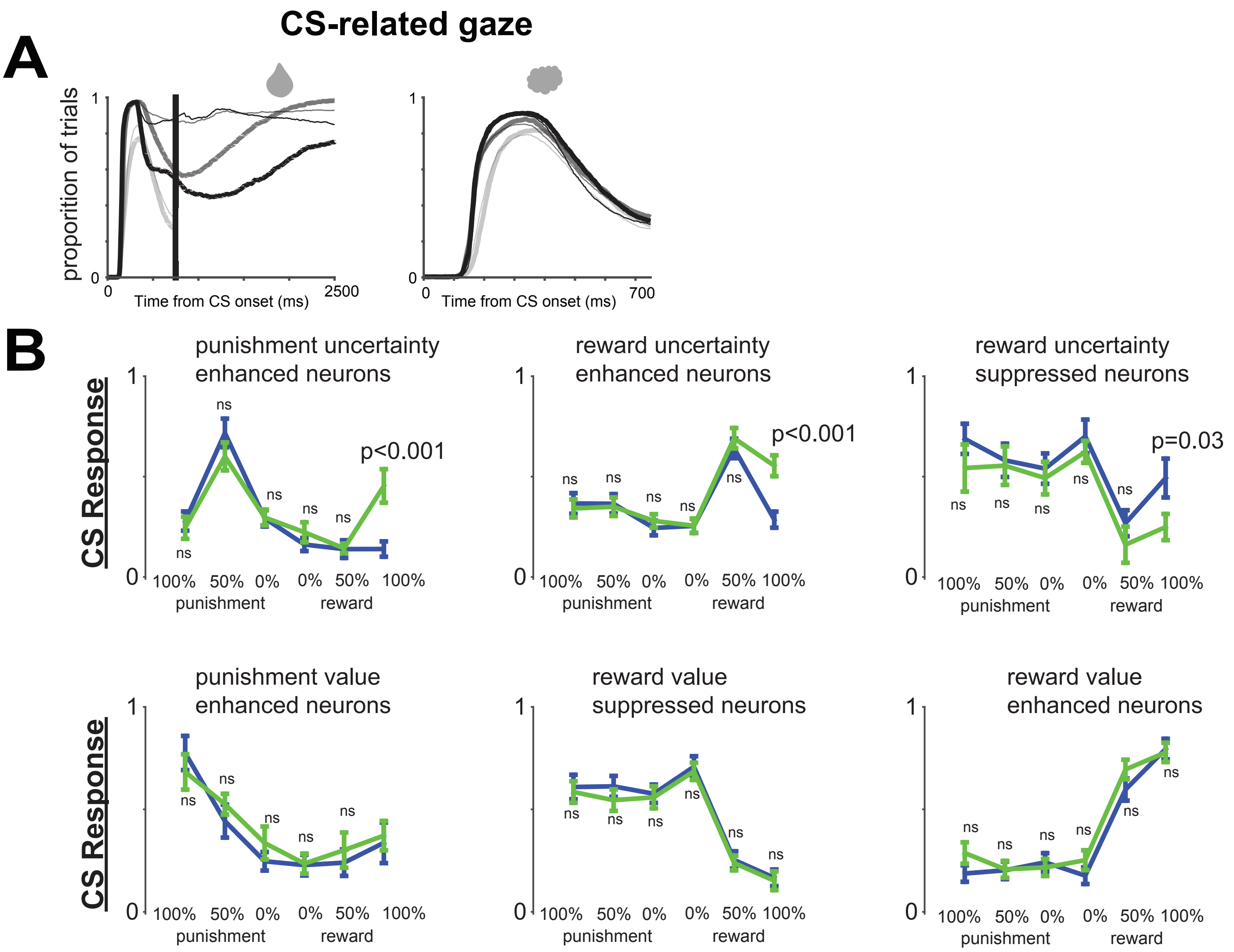

***Supplementary Figure 10* – Behavioral and neuronal differences across blocks with and without abort cues.** (**A**) Gaze behavior is modulated by uncertainty and by abort possibility. Thick lines show behavior during blocks in which abort cues were never presented. Thin lines show behavior during blocks in which abort cues were presented 50 % of the time. Lines are colored as in Figure 1. Thick line at 750 ms following CS onset shows the time during which the abort cue would have been presented. Because the monkeys often aborted the non-rewarding trials and blinked during the later epochs of punishment-possible trials (Figure 1), we show behavior during 0% reward trials and 100, 50, and 0% punishment trials until the time of the abort cue would have been presented. After the time of the abort cue, we show behavior during the 100% and 50% reward trials in which monkeys did not abort. The monkeys could have looked away from the monitor to minimize the probability of error saccades after reward CSs were presented towards the abort cues. Instead, their overt attention increased towards reward CSs (100 and 50%). (**B**) Neuronal activity before abort cues were presented (750 ms window following CS onset, before the abort cue) during blocks of trials in which abort cues were never presented (blue) versus blocks of trials in which the abort cue appeared during half of the trials (green) for 6 groups of ACC CS modulated neurons. Consistent differences were observed for uncertainty-sensitive neurons following 100% reward CSs. Before averaging, single neurons' CS responses were normalized to the maximum CS response across 12 CS conditions; from 0 to 1. Error bars represent SEM. Ns – not significant (paired sign rank test; p>0.05).

**Valence-specific**, n = 164 / 329 **49.9%**		**Valence non-specific**, n = 45 / 329 **14%**	
**Reward value** n = 92 / 329	**Punishment value** n = 72 / 329	**Intensity** n =32 / 329	**General Value** n = 13 / 329
Monkey 1, n=21/170<br>Monkey 2, n=71/159	Monkey 1, n=60/170<br>Monkey 2, n=12/159	Monkey 1, n=22/170<br>Monkey 2, n=10/159	Monkey 1, n=7/170<br>Monkey 2, n=6/159

***Supplementary Table 1* – Summary of neurons' correlations with predictions of reward and punishment during the CS epoch.** Data for single cells is shown in Supplementary Figure 3A. Neuronal categories are based on Figure 1C.

	Reward (+)		Reward (-)		Punishment (+)		Reward uncertainty		Punishment uncertainty	
Time spent blinking	0	0	3.571429	0	0	0	0	0	4.347826	0
Pupil response	0	0	0	0	0	0	1.754386	1.754386	0	0
Pupil during ITI	0	0	0	0	0	0	0	1.754386	0	0
Time spent licking	0	0	0	0	0	0	0	0	0	4.347826
	0	0	0	0	0	0	0	0	0	0
Object selectivity	6.66667		0		0		8.77193		0	

***Supplementary Table 2* – Summary of trial-by-trial relationships of ACC neurons with conditioned responses**. Neurons were categorized into types (top of table) based on the results of Figures 2 and 5. The percentage of neurons in each category that displayed trial-by-trial correlations with conditioned responses (left) is reported. Positive correlations are colored red (left column of each category), negative are colored in blue (right column). Below, percentage of neurons that displayed a difference in their responses to two different visual fractal objects that conveyed the same outcome probability is reported. Significance thresholds are $p<0.01$.
